# How do word valence and classes influence lexical processing? Evidence from virtual reality emotional contexts

**DOI:** 10.3389/fpsyg.2022.1032384

**Published:** 2023-01-06

**Authors:** Xiaoying Wang, Sumin Zhang, Xiaohuan Zhang

**Affiliations:** ^1^Wu’an Comprehensive Vocational Education Center, Wuan, Hebei, China; ^2^School of Foreign Studies, Zhejiang Gongshang University, Hangzhou, Zhejiang, China; ^3^Mental Health Education Center, Guizhou Forerunner College, Huishui, Guizhou, China

**Keywords:** VR emotional context, word class, word valence, emotional words processing, galvanic skin responses

## Abstract

The current study examines the influence of word class (i.e., noun vs. adjective) and valence (i.e., positive vs. negative vs. neutral) on the processing of emotional words under different virtual reality (VR) emotional contexts. To this end, 115 participants performed a modified affect labeling task after experiencing different VR scenarios. Their galvanic skin responses were also examined to further gauge the different effects of VR contexts. The results demonstrated significant main effect for word valence, indicating more processing of positive words relative to neutral words which are processed more than negative words. The results also demonstrated significant main effect for word class, indicating more processing of nouns in contrast to adjectives. Additionally, the results indicated that both positive and negative VR contexts could stimulate participants to select more positive words though negatively valenced words were processed more under negative VR context relative to positive VR context. However, the amplitude of galvanic skin responses in positive VR was lower than that in negative VR. The results were interpreted in line with the situation-consistency effects, the mood-consistency effects, the specific nature of VR context, and the different features of different word classes in terms of concreteness, imageability, arousal, and valence.

## Introduction

Effects of emotional valence (positive vs. negative vs. neutral) have witnessed burgeoning attention in lexical processing research. While it is generally suggested that valence play a crucial role in lexical processing, the bulk of the previous studies have been prominently featured by discrete emotional stimuli limited to emotional face pictures or sentences (Wieser et al., 2014; Diéguez-Risco et al., 2015; Fan et al., 2018; Li et al., 2020) or lexical decision tasks (Barriga-Paulino et al., 2022) under laboratory settings. In addition, nouns and adjectives are different in their concreteness and imageability (Peti-Stantić et al., 2021), semantic references (Xia et al., 2016), and processing mechanism (Zhu et al., 2011; Kolbeneval and Alexandrov, 2016; Zhang, 2019a), which could modulate an individual’s lexical processing with different valences under different contexts. Emotional experiences are ‘ingredients’ in the creation of emotional perceptions (Glenberg et al., 2009; Lindquist, 2017; Zhang, 2019a,b), and evidence from controlled laboratory settings cannot gauge fully an individual’s real emotional experiences and their effects on emotional words processing (Pavlenko, 2008). Therefore, it is highly necessary to explore emotional words processing under real emotional contexts. Virtual reality digital technology has a lot to offer to an individual’s enhanced experience and cognition because of its immersive and authentic/real-life environment (Parsons, 2015; Roy et al., 2019) which could affect the execution of motor responses (Sell and Kaschak, 2011; Zhang and Zhang, 2015). Given that galvanic skin responses are important indicators of an individual’s stimulated responses (Bai and Yue, 2013), it is also necessary to explore whether there are some changes in an individual’s galvanic skin responses under different VR contexts. In light of these, using VR technology, this study intends to assess the influence of word valence and classes on emotional words processing and to examine the galvanic skin responses under different VR emotional contexts. Research into this domain can allow us to resolve at least some of the empirical paucity in the literature in light of the role of VR emotional context in human experience and in the processing of emotional words with different word classes (Ni and Jin, 2020).

## Research background

### The modulation role of word class in the effect of valence on lexical processing

Valence effects can be modulated by word classes because different word classes have different emotional arousal and processing mechanisms for an individual (Kolbeneval and Alexandrov, 2016; Zhang, 2019a). Nouns, verbs, and adjectives are different in their concreteness, imageability, and semantic references, indicating different complexity in recognition and processing (Gleitman, 1990; Xia et al., 2016; Peti-Stantić et al., 2021). Some studies even found that different word classes were associated with different neurons, and thus had different stability, consistency and gradability (Waxman and Booth, 2001; Kolbeneval and Alexandrov, 2016). While some study further verified that nouns had processing advantages over others due to their concreteness (Zhang and Zhang, 2015), some study found that adjectives could have processing advantages over nouns and verbs (Zhang, 2019a). Still some study found that compared to verbs, nouns could be more prone to be influenced by extreme emotions, and positive emotion could enhance the nouns’ processing advantages (Zhu et al., 2011). Given the inconsistent findings in the processing of different word classes, we directed our research to the role of word class in the effects of valence on emotional words processing. In addition, on the basis of the strong evidence that emotion information processing has self effects that cannot be separated from human’s body and mind (Boroditsky and Ramscar, 2002; Pavlenko, 2008; Zhang, 2019b) and that embodied simulation depth can influence an individual’s lexical processing (Sell and Kaschak, 2011; Zhang and Zhang, 2015), it is possible that the role of word class in the effects of valence on an individual’s physiological responses may be influenced by different environmental experiences. However, there is a paucity of studies to directly address the effects of word class on the processing of words with different valences under different environments. Thus, it remains unclear whether the context effects are driven primarily by differences in valence, or by differences in word class. Therefore, the present study was interested not only in whether valence effects would be observed differently for different word classes, but also in whether different effects would be evident in different contexts.

Researches carried out in laboratory settings have well established the role of valence in lexical processing, suggesting that words with different emotional valence could be different in their processing. However, the findings are far from universal. The majority of previous studies have found that negatively valenced words, compared to those with positive or neutral valence, could exert more processing in quantity, intensity, and neural responses (e.g., Eilola et al., 2007; Kennedy and Most, 2015; Kazanas and Altarriba, 2016). Using Stroop paradigm, Eilola et al. (2007) investigated the Finnish-English bilinguals’ emotional words processing and found that motional activation in both L1 and L2 was more for the negative and taboo words than for the neutral and positive stimuli. Similar support was provided by Kennedy and Most (2015) who also found that negative stimuli could exert more effects on an individual’s physiological and psychological responses relative to neutral stimuli, and accordingly could automatically capture the individual’s attention and resulted in attentional bias. Using unmasked priming paradigm, Kazanas and Altarriba (2016) compared the processing of emotion-label words (e.g., happy, sad) and emotion-laden words (e.g., wedding, funeral) and echoed the previous studies. In contrast to the aforementioned studies that stressed the advantage of negative valence stimuli, a number of studies have indicated rather complicated findings using various tasks. Kousta et al. (2009, p. 473) argued that emotional stimuli, irrespective of their polarity, could capture more attention than neutral stimuli “due to the relevance of both negative and positive stimuli for survival and for the attainment of goals.” El-Dakhs and Altarriba (2019) examined the influence of word valence on the processing of emotional words among Arabic-English bilinguals, and found results yielded from free recall and rating tasks generally supported the influence of word valence on the processing of emotional words, whereas results from word association task did not seem to support the influence of word valence. Mixing results were also revealed by Crossfield and Damian (2021) because a significant processing advantage for positive words over negative and neutral words was found in the lexical decision task, whereas in the emotional Stroop task valence alone exerted no significant effects. The different findings in different studies as measured by various tasks lend further support to the complexity of valence effects in emotional words processing, indicating potential confounding factors other than word class in the valence effects on lexical processing.

The discrete, context-detached stimuli shared by all the previous tasks might explain the rather conflicting findings as revealed by the aforementioned studies because emotion, context and experienced cognition are closed related (Lindquist, 2017; Wu et al., 2020). In agreement with this premise, additional studies have tried to characterize context effects on emotional word processing. Using compound stimuli that consisted of words superimposed on pictures of affective faces, Stenberg et al. (1998) found that the speed advantage for positively valenced words could be modified by affective faces. Additionally, the negatively valenced words were facilitated when shown with a negative emotion face, and were inhibited when shown with a positive emotion face. The effects of facial expressions on the processing of emotional words with different valence have also been supported by Diéguez-Risco et al. (2015) who found that situational context could modulate the processing of facial expressions of emotion, indicating significant congruency effects. Further evidence comes from Lindquist (2017) who found that linguistic context could facilitate participants to recognize facial expressions more quickly and correctly. Li et al. (2020) also explored the role of language context in facial expressions processing and recognition, and their findings generally confirmed the linguistic context effects on the judgment of facial expressions. However, different from other studies, they also found that valence of neutral faces was significantly higher in positive than in negative contexts, and the arousal of neutral faces in the self-related context were significantly higher as compared to the other-related context.

One corollary of the aforementioned relation between emotion and cognition is that linguistic contexts likely impinge on emotional words processing, and that contextual effects may be different for different words. However, what should be noted is that the emotion-cognition relation has been mainly explored within a controlled laboratory setting where emotion invoking stimuli were exposed very briefly. It is still unclear regarding how well stationary laboratory behavior predicts task performance when the stimuli have features more typical of real-world environments (Pavlenko, 2008; Roy et al., 2019). Therefore, the potential differences between laboratory settings and real environments call for further research to address the effects of real emotional contexts on lexical processing.

### Virtual reality simulated scenarios and galvanic skin responses

Virtual reality digital technology can enable its users to be immersed in a 3D virtual environment, and accordingly activate active interaction between the simulated reality and users. Virtual reality can also allow for the use of multiple senses that function simultaneously during information processing (Roy et al., 2019). In a VR context, the addition of living objects such as virtual people or animals can more realistically imitate the real world, so users could have a virtual sense of immersion (Fisher and Fraser, 1998). Additionally, the spatial presence and the social presence evoked by VR technology can enhance a user’s environmental experience, which, in turn, can inspire greater interaction with the multimedia system and more stimulated responses (Bian et al., 2015). Language construction is inseparable from emotional experience, and context is an important factor that could affect emotional words perception and processing (Zhang, 2019a; Bonfieni et al., 2020). Thus, VR can provide a potentially real emotional context for lexical processing studies.

In addition to a potentially real emotional context, VR can also create an enhanced self-related context which is a prominent factor in an individual’s emotion information processing (Meshi et al., 2016). An individual’s emotional perception and processing originates from his/her own emotion experience, and different experiences can induce different emotional and physiological responses (Centerbar et al., 2008). It has been proved that self-related information was associated with more emotional arousal because it could be recognized and processed more easily (Kim, 2012). Therefore, VR enhanced context could bring in enhanced interaction, and accordingly could create enhanced emotional experiences different from the controlled laboratory settings (Roy et al., 2019). Emotional cognition is constructed through a person’s experience and interaction with the environment (Glenberg et al., 2009); thus, it is interesting to investigate whether the enhanced self-related context can yield similar results to the previous laboratory studies. Furthermore, negative emotions were reported to be positively related to anxiety (Roy et al., 2019) and negatively related to mindfulness meditation (Morgan and Jonah, 2021). Thus, it is also necessary to investigate whether an individual’s emotional words processing can be different when experiencing different VR simulated environments. This direction of research can help us better understand how we perceive, interpret, and react to the different events and how to intervene our psychological stress with VR (Zhang et al., 2019).

Together with these predictions for physiological responses, we also hypothesize that VR simulated emotional environments may also influence an individual’s galvanic skin responses because from which we can observe an individual’s stimulated responses under different circumstances (Bai and Yue, 2013). A stressful event can increase rapidly our heart rate, elevate our skin temperature, and heighten our memory for the experience (Kemeny, 2003). Therefore, the analyses of galvanic skin responses, together with psychological responses, can contribute to rapid coordination for perception and action, which is essential to react correctly to unexpected and dynamic events (Roy et al., 2019). However, to the best of our knowledge, no existing studies have addressed an individual’s galvanic skin response under different VR simulated contexts. Thus, galvanic skin responses elicited from different VR contexts can further add to the growing literature that emotional context studies can broad “the scope beyond the laboratory for extended validity and generalizability to real-world outcomes” (Schmälzle et al., 2017; Roy et al., 2019, p. 11).

In sum, the current study constitutes a new attempt to assess the influence of word valence and classes on lexical processing under VR simulated emotional contexts. This attempt represents a significant contribution due to the scarcity of research in this perspective. The current study gains additional significance from a number of factors. First, the current study examines valence and emotional context effects among different word classes, which will help understand any potential influence for different concreteness and imageability. Secondly, the effects of VR simulated emotional contexts on emotional words are largely underrepresented in lexical processing research. Findings from this perspective may prove specifically significant because VR features more typical of real-world environments and can heighten immersive experience (Parsons, 2015; Schmälzle et al., 2017). Specifically, the current study addresses the following research questions:

1. Are there any differences between the positive and negative VR contexts in the processing of differently valenced words with different word classes?
2. Are there any differences in an individual’s galvanic skin responses under different VR contexts?

## Research design

### Participants

One hundred and fifteen participants were recruited from a vocational college. All participants were 2nd graders (20 males and 95 females) whose average age was 20.05 (SD = 0.96). All participants were healthy, reported no history of fear of height, cardiovascular diseases, psychiatric illness, or neurologic disorder. Additionally, all participants had normal or corrected-to-normal vision and were right-handed, and participated in the experiment voluntarily and received a certain reward after the experiment. Consent form were signed by the participants’ representative, and all participants were free to retreat from the experiment whenever they wanted. Ethics approval were also checked and approved by Academy Department of the college.

### Stimuli

There were 60 emotional word stimuli, including 30 nouns (10 positive + 10 negative + 10 neutral) and an equal number of adjective counterparts (10 positive + 10 negative + 10 neutral). According to Fan et al. (2018), all the emotional words were two-character words picked out from Chinese affective words system (CAWS) whose frequency ranged from 20 to 90. For instance, “爱情” (àiqíng: love), “宫廷” (gōngtíng: palace) and “厕所” (cèsǔo: toilet); “卑贱”(bēijiàn: lowly), “单独” (dāndú: alone), and “勇敢” (yǒnggǎn: brave). Following Barriga-Paulino et al. (2022), the valence of all the positively valenced words is ≥7, the valence of all the neutral words is between 4 and 6, and the valence of all the negatively valenced words is <3. The 60 emotional words formed a randomly arranged word list from which all participants in the formal experiment were asked to select after experiencing different VR scenarios.

Results of the repeated measures ANOVA showed significant differences between positively, neutrally, and negatively valenced nouns in term of the valences (*M* = 7.36, SD = 0.27; *M* = 5.38, SD = 0.40; *M* = 2.73, SD = 0.48; *F*_(2,18)_ = 465.43, *p* < 0.001, *η*^2^ = 0.981), indicating significantly higher valence of positively nouns relative to neutrally valenced nouns (Mean difference = 1.98, *p* < 0.001) and negatively nouns (Mean difference = 4.63, *p* < 0.001), and significantly higher valence of neutrally valenced nouns relative to negatively valenced nouns (Mean difference = 2.65, *p* < 0.001). Significant differences were also shown between positively, neutrally, and negatively valenced nouns in term of arousal (*M* = 6.42, SD = 0.64; *M* = 3.93, SD = 0.40; *M* = 6.01, SD = 0.94; *F*_(2,18)_ = 28.91, *p* < 0.001, *η*^2^ = 0.763), indicating significantly higher arousal of positively and negatively valenced nouns relative to neutrally valenced nouns (Mean difference = 2.49, *p* < 0.001; Mean difference = 2.08, *p* < 0.001) though insignificant differences were found between negatively and neutrally valenced nouns (Mean difference = 0.40, *p* = 0.393). However, no significant differences were showed between positively, neutrally, and negatively valenced nouns in terms of the frequency (*M* = 37.40, SD = 29.08; *M* = 29.90, SD = 24; *M* = 21.80, SD = 26.02; *F*_(2,18)_ = 1.27, *p* = 0.305, *η*^2^ = 0.124). Neither were significant differences found between negatively, positively, and neutrally valenced nouns in terms of the familiarity as shown by pilot participants’ self-report (*M* = 6.997, SD = 0.018; *M* = 6.993, SD = 0.025; *M* = 6.997, SD = 0.018; *F*_(2,58)_ = 0.244, *p* = 0.785, *η*^2^ = 0.008).

Results of the repeated measures ANOVA also showed significant differences between positively, neutrally, and negatively valenced adjectives in term of the valences (*M* = 7.19, SD = 0.12; *M* = 4.60, SD = 0.41; *M* = 2.37, SD = 0.25; *F*_(2,18)_ = 660.82, *p* < 0.001, *η*^2^ = 0.987), indicating significantly higher valence of positively adjectives relative to neutrally valenced adjectives (Mean difference = 2.59, *p* < 0.001) and negatively valenced adjectives (Mean difference = 4.82, *p* < 0.001), and significantly higher valence of neutrally valenced adjectives relative to negatively valenced adjectives (Mean difference = 2.23, *p* < 0.001). Significant differences were also shown between positively, neutrally, and negatively valenced adjectives in term of arousal (*M* = 6.53, SD = 0.32; *M* = 5.39, SD = 0.99; *M* = 6.74, SD = 0.33; *F*_(2,18)_ = 12.35, *p* < 0.001, *η*^2^ = 0.579), indicating significantly higher arousal of positively and negatively valenced adjectives relative to neutrally valenced adjectives (Mean difference = 1.14, *p* = 0.017; Mean difference = 1.35, *p* = 0.001) though insignificant differences were found between negatively and neutrally valenced adjectives (Mean difference = 0.21, *p* = 0.158). However, no significant differences were showed between positively, neutrally, and negatively valenced adjectives in terms of the frequency (*M* = 38.67, SD = 17.59; *M* = 24.78, SD = 13.26; *M* = 21.33, SD = 12.16; *F*_(2,18)_ = 3.02, *p* = 0.077, *η*^2^ = 0.274). Neither were significant differences found between negatively, positively, and neutrally valenced adjectives in terms of the familiarity as shown by pilot participants’ self-report (*M* = 6.990, SD = 0.031; *M* = 6.990, SD = 0.031; *M* = 6.993, SD = 0.025; *F*_(2,58)_ = 0.121, *p* = 0.886, *η*^2^ = 0.004).

As to the VR digital technology used, VR headset model was HTC VIVEProt, and headset parameters were two 3.5-inch AMOLED screens with 1,440 × 1,600single eye resolution and 2,880 × 1,600 binocular resolution; The refresh rate was 90 Hz, and the field of view was 110 degrees; The audio output included Hi-Res Audio certified headset and Hi-Res Audio certified earset (detachable); Sensors included SteamVR tracking technology, G-sensor correction, gyroscope gyroscope, proximity sensor, and eye comfort Degree setting (IPD); The control panel was MI PAD4, and the processor was eight-core 2.20 GHz; As to the computer model, the processor was i7-8,700 CPU, 3.20 GHz, and the system model was a 64-bit operating system.

As to the VR context, two types were designed: Positive scenario and negative scenario. In the negative scenario VR elevator design, there were 101 floors in the elevator. Influenced by the differences in sweat glands and body glands of the participants, the elevator was set to accelerate and shake 3–5 times during the ascent to produce a falling effect, which was designed to prompt the participants to show significant changes in their heart rate. The duration of the whole experiment including the preparation time was about 3 min and 20–26 s. In the positive scenario VR elevator design, the elevator did not go up, and around the participants there would appear an endless prairie where elephants and other animals were walking, grazing, or playing leisurely. At the same time, soothing and soft music was played, accompanied by instructions such as “take a deep breath, relax, take a deep breath, and relax,” trying to create a relaxed and peaceful atmosphere. The duration of the whole experiment including the preparation time is also about 3 min 20–26 s. The two VR scenarios’ valence and arousal were classified on a 7-point rating scale by participants from the pilot experiment. Results of the *T*-test showed significant differences between positively and negatively valenced VR scenarios in terms of the valences (*M* = 5.93, SD = 0.64; *M* = 2.87, SD = 0.57; *T*_(29)_ = 26.26, *p* < 0.001) and insignificant differences in terms of the arousal though that of the negative VR context was much higher (*M* = 4.37, SD = 0.56; *M* = 4.57, SD = 0.68; *T*_(29)_ = −1.80, *p* = 0.083).

### Procedure

**Figure 1:**
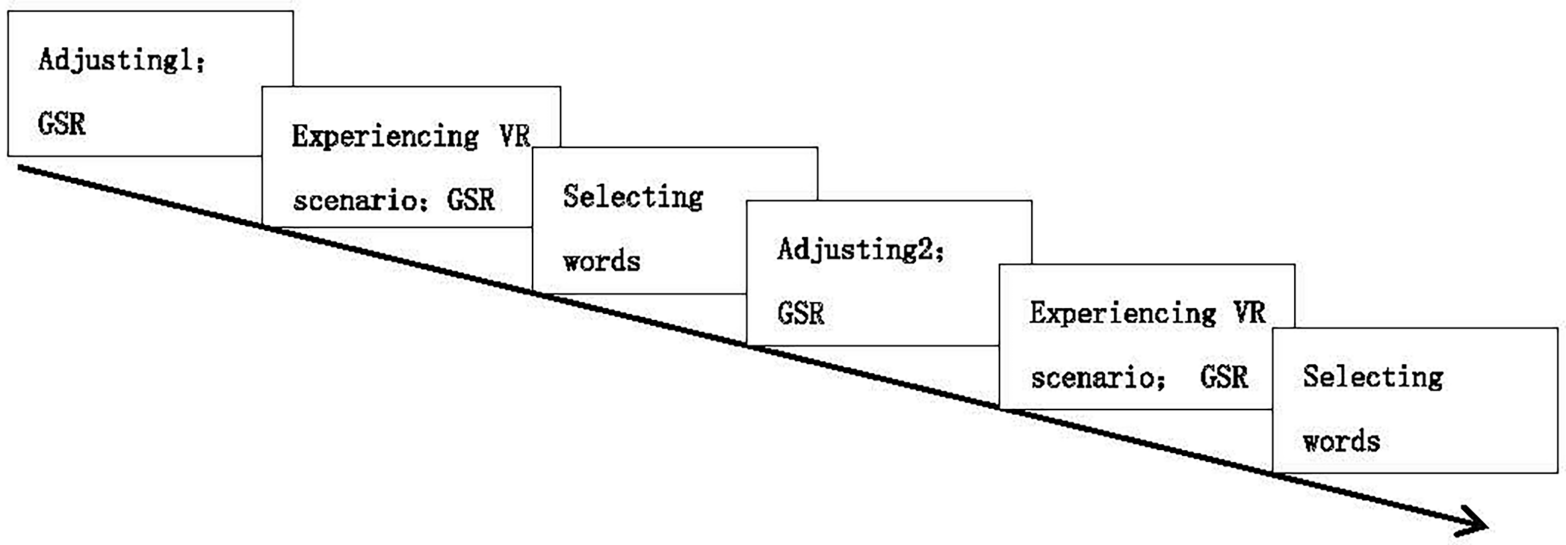
VR elevator experiment procedure. GSR, galvanic skin responses.

The elevator experiment adopted a 2 (context type: positive context, negative context) × 3 (word valence: positive, negative, neutral) × 2 (word class: noun, subjective) within-subject design. The independent variables were word class, word valence, and VR context, and the dependent variables were the number of differently valenced nouns and adjectives selected by participants and the galvanic skin responses. For details, see the flowchart (Figure 1).

The experiment was carried out on participants one by one with the help of two colleges whose major was behavioral science and cognitive psychology, respectively. All participants were asked to put on their headset before experiencing the negative and positive VR scenarios, and would be given about 1 min to experience a VR prairie scenario or a snow mountain scenario to relax to slow down their heart rate. The specific time to slow down the heart rate was based on the specific heart rate characteristics of the participants. Using Latin square experimental design, some participants (*n* = 50) were randomly selected to experience the positive VR scenario first and then the negative VR scenario, and the other part of the participants (*n* = 65) were asked to experience the negative VR scenario first and then the positive VR scenario in order to balance order effects.

Post-experience conditioned data of participants under different VR scenarios were collected using the adapted affect labeling task, following Lieberman et al. (2011) and Yue et al. (2020). Affect labeling refers to using emotional words to name emotional stimuli or the emotions evoked by them. Namely, the affect labeling task requires the participants to process facial emotions and name them with emotional words, such as “anger,” “joy,” etc. (Yue et al., 2020, p. 31). In this study, immediately after experiencing the positively and negatively VR scenarios, all participants were asked to tick words as quickly as possible within 1 min from a given word list which contained 60 emotional words (see Appendix 1). The ticking time was set according to the time used by recruited students in the pilot experiment. Totally, there were 60 emotional words in the given word list, including 10 positively, 10 negatively, and 10 neutrally valenced nouns, and an equal number of emotionally identical adjective counterparts. All words were arranged randomly on a response sheet which the participants had been provided with to perform the task. Additionally, potential confounding factors like semantic relatedness between the scenarios and words were also ruled out through a careful examination of the words in the word list and the two VR scenarios by all the authors of the present study. In addition, the pilot experiment showed that in general, the scenarios and words were not semantically related as measured by a yes/no judgment task which required the participants to judge whether meanings of the words in general were related to the VR scenarios experienced (Frequency_yes_ = 5, Frequency_no_ = 25; loglikelihood = 14.56, *p* = 0.0001). In order to further test the semantic relatedness between the scenarios and words, a post-experiment norming study was done using a 7-point rating task among 30 participants recruited from the same college (Minimun = 1; Maximum = 1.13; *M* = 1.07, SD = 0.03). Both results indicated a rather low semantic relatedness between the two.

Data collection of galvanic skin responses was implemented with reference to Bai and Yue (2013, p. 718). After wiping the electrode with 75% medical alcohol, the Ag/AgC1 electrodes were wrapped around the end pulps of the index finger and ring finger of the participants’ left hand, respectively. Electrodes were connected to the GSR100C module of the physiological recorder to record the electrodermal activity with a sampling rate of 200 Hz. 200 magnifications. The galvanic skin responses were collected before and during experiencing the positive VR scenario and the negative VR scenario.

Before the formal study, 30 students from the same college were recruited and required to pick out the words that could describe the emotion evoked by the scenario experienced from a word list, and then were asked to perform a 7-point rating scale to obtain the information about familiarity level of the emotional words and valences of the two VR scenarios. These students were different from the participants in the formal experiment. After experiencing the 1st VR scenario and selecting words from a given word list, all participants from the pilot and the formal experiment were required to have about 5 min’ rest. Upon task completion, the response sheets provided to participants to perform the task were collected immediately.

## Data analysis

### Analysis of the number of words selected under different virtual reality contexts

A 2 (context type: positive VR scenario and negative VR scenario) × 3 (word valence: positive, negative and neutral) × 2 (word class: noun and adjective) repeated-measures ANOVA was performed on words selected by participants under different VR scenarios (see Table 1). The main effect for word valence was significant (*F*_(2,456)_ = 272.26, *p <* 0.001, *η^2^* = 0.544), indicating more processing of positively valenced words relative to neutral words (*M* = 3.36, SD = 0.13; *M* = 1.67, SD = 1.10; *p* < 0.001) and more processing of neutral words compared to negatively valenced words (*M* = 1.67, SD = 1.10; *M* = 0.51, SD = 0.06, *p* < 0.001). The main effect for word class was also significant (*F*_(1,228)_ = 23.72, *p <* 0.001, *η^2^* = 0.094), indicating more processing of nouns in contrast to adjectives (*M* = 2.02, SD = 0.08; *M* = 1.68, SD = 0.06, *p* < 0.001). Additionally, there were significant two-way interactions between valence and context (*F*_(2,456)_ = 103.24, *p* < 0.001, *η^2^* = 0.312), approximately significant two-way interactions between word class and context (*F*_(1,228)_ = 3.73, *p* = 0.055, *η^2^* = 0.016), and significant two-way interactions between valence and word class (*F*_(2,246)_ = 45.65, *p* < 0.001, *η^2^* = 0.167). Furthermore, there were also significant three-way interactions between context, valence, and word class (*F*_(2,456)_ = 10.34, *p* < 0.001, *η^2^* = 0.043).

**Table 1:** Repeated-measures three-way ANOVA of valence, word class, and context.

Variables	*F*	*p*-value	*η^2^*
Valence	272.26***	<0.001	0.544
Word class	23.72***	<0.001	0.094
Valence * context	103.24***	<0.001	0.312
Word class * context	3.73*	0.055	0.016
Valence * word class	45.65***	<0.001	0.167
Valence * word class * context	10.34***	<0.001	0.043

* The significant level is 0.05.

*** The significant level is 0.001.

The simple effect analysis indicated significant interference between the three variables in term of word class (see Table 2).

**Table 2:** Simple effects analyses of words selected in term of word class.

VR context	Valence	(*I*) word class	(*J*) word class	(*I*-*J*)	*p*-value
Positive	Positive	Noun	Adjective	1.09***	<0.001
	Negative	Noun	Adjective	−0.35**	0.002
	Neutral	Noun	Adjective	0.67***	<0.001
Negative	Positive	Noun	Adjective	1.06***	<0.001
	Negative	Noun	Adjective	−0.13	0.247
	Neutral	Noun	Adjective	−0.32*	0.025

* The significant level is 0.05.

** The significant level is 0.01.

*** The significant level is 0.001.

Under positive VR context, (1) for positively valenced words, significant differences were found between nouns and adjectives (*M*_positive nouns_ = 3.50, SD = 0.23; *M*_positive adjectives_ = 2.41, SD = 0.17; *p* < 0.001), indicating more processing of positively valenced nouns as compared to adjectives; (2) for negatively valenced words, significant differences were found between nouns and adjectives (*M*_negative nouns_ = 0.32, SD = 0.09; *M*_negative adjectives_ = 0.67, SD = 0.11; *p* = 0.002), indicating more processing of negatively valenced adjectives as compared to nouns; (3) for neutral words, significant differences were also found between nouns and adjectives (*M_neutral nouns_* = 3.287, SD = 0.165; *M_neutral adjectives_* = 2.617, SD = 0.140), indicating more processing of neutral nouns as compared to adjectives. Overall, under positive VR context, the processing of positively and neutrally valenced nouns was significantly more than that of positively and neutrally valenced adjectives, and the processing of negatively valenced adjectives was significantly more than that of negatively valenced nouns.

Under negative VR context, (1) for positively valenced words, significant differences were found between nouns and adjectives (*M*_positive noun_ = 4.30, SD = 0.23; *M*_positive adjective_ = 3.24, SD = 0.17; *p* < 0.001), indicating processing advantages of positively valenced nouns over adjectives; (2) for negatively valenced words, no significant differences were found between nouns and adjectives (*M*_negative noun_ = 0.46, SD = 0.09; *M*_negative adjective_ = 0.59, SD = 0.11; *p* = 0.247), indicating similar processing of negatively valenced adjectives and nouns; (3) for neutral words, significant differences were also found between nouns and adjectives (*M_neutral noun_* = 0.24, SD = 0.17; *M_neutral adjective_* = 0.56, SD = 0.14; *p* = 0.025), indicating processing advantages of neutral adjectives over nouns. In a word, under negative VR context, the processing of positively valenced nouns was significantly more than that of positively valenced adjectives, the processing of negatively valenced nouns and adjectives was similar to each other, and the processing of neutral adjectives was significantly more than that of neutral nouns.

Together, the results as shown in Table 2 demonstrate that while the processing advantages of positively valenced nouns over positively valenced adjectives was showed under both positive and negative VR contexts, the processing differences between negatively and neutrally valenced nouns and adjectives were different under different VR contexts: Positive VR could stimulate more processing of neutral nouns and negative valenced adjectives, whereas negative VR could stimulate more processing of neutral adjectives.

The simple effect analysis indicated significant interference between the three variables in term of valence (see Table 3).

**Table 3:** Simple effects analyses of words selected in term of valence.

VR context	Word class	(*I*) valence	(*J*) valence	(*I*-*J*)	*p*-value
Positive	Noun	Positive	Negative	3.18***	<0.001
			Neutral	0.21	0.346
		Neutral	Negative	2.97***	<0.001
	Adjective	Positive	Negative	1.74***	<0.001
			Neutral	−0.21	0.255
		Neutral	Negative	1.95***	<0.001
Negative	Noun	Positive	Negative	3.84***	<0.001
			Neutral	4.06***	<0.001
		Neutral	Negative	−0.23	0.228
	Adjective	Positive	Negative	2.65***	<0.001
			Neutral	2.68***	<0.001
		Neutral	Negative	−0.04	0.839

*** The significant level is 0.001.

Under positive VR context, (1) for nouns, significant differences were found between positively and negatively valenced nouns (*M*_positive nouns_ = 3.50, SD = 0.23; *M*_negative nouns_ = 0.32, SD = 0.09; *p* < 0.001) and between neutrally and negatively valenced nouns (*M*_neutral nouns_ = 3.29, SD = 0.17; *M*_negative nouns_ = 0.32, SD = 0.09; *p* < 0.001), whereas no significant differences were found between positively valenced nouns and neutral nouns (*M*_positive nouns_ = 3.50, SD = 0.23; *M*_neutral nouns_ = 3.29, SD = 0.17; *p* = 0.346), indicating more processing of positively and neutrally valenced nouns as compared to negatively nouns; (2) for adjectives, significant differences were found between positively and negatively valenced adjectives (*M*_positive adjectives_ = 2.41, SD = 0.17; *M*_negative adjectives_ = 0.67, SD = 0.11; *p* < 0.001) and between neutrally and negatively valenced adjectives (*M*_neutral adjectives_ = 2.62, SD = 0.14; *M*_negative adjectives_ = 0.67, SD = 0.11; *p* < 0.001), whereas no significant differences were found between positively valenced adjectives and neutral adjectives (*M*_positive adjectives_ = 2.41, SD = 0.17; *M*_neutral adjectives_ = 2.62, SD = 0.14; *p* = 0.255), indicating more processing of positively and neutrally valenced adjectives as compared to that of negatively valenced adjectives. In sum, under positive VR context, the processing of positively and neutrally valenced nouns and adjectives was significantly more than that of negatively valenced nouns and adjectives while no significant differences were found between positively and neutrally valenced nouns and adjectives.

Under negative VR context, (1) for nouns, significant differences were found between positively and negatively valenced nouns (*M*_positive nouns_ = 4.30, SD = 0.23; *M*_negative nouns_ = 0.46, SD = 0.09; *p* < 0.001) and between positively and neutrally valenced nouns (*M*_positive nouns_ = 4.30, SD = 023; *M*_neutral nouns_ = 0.24, SD = 0.17; *p* < 0.001), whereas no significant differences were found between negatively and neutrally valenced nouns (*M*_negative nouns_ = 0.46, SD = 0.09; *M*_neutral nouns_ = 0.24, SD = 0.17; *p* = 0.228); (2) for adjectives, significant differences were found between positively and negatively valenced adjectives (*M*_positive adjectives_ = 3.24, SD = 0.17; *M*_negative adjectives_ = 0.59, SD = 0.11; *p* < 0.001) and between positively and neutrally valenced adjectives (*M*_positive adjectives_ = 3.24, SD = 0.17; *M*_neutral adjectives_ = 0.56, SD = 0.14; *p* < 0.001), whereas no significant differences were found between negatively and neutrally valenced adjectives (*M*_negative adjectives_ = 0.59, SD = 0.11; *M*_neutral adjectives_ = 0.56, SD = 0.14; *p* = 0.839). In general, under negative VR context, the processing of positively valenced nouns and adjectives was significantly more than that of negatively and neutrally valenced nouns and adjectives, whereas no significant differences were found between neutrally and negatively valenced nouns and adjectives.

Together, the results as shown in Table 3 demonstrate that while positive VR context indicated processing advantages of positively and neutrally valenced nouns and adjectives over negatively valenced nouns and adjectives, negative VR context showed processing advantages of positively valenced nouns and adjectives over neutrally and negatively valenced nouns and adjectives. The results also demonstrate insignificant processing differences between neutrally and positively valenced nouns and adjectives under positive VR context, and insignificant processing differences between neutrally and negatively valenced nouns and adjectives under negative VR context. Generally speaking, these findings indicate that (1) both positive and negative VR contexts could stimulate participants to select more positive words, irrespective of word classes though negatively valenced words were processed more under negative VR scenario than under positive VR scenario, and that (2) the processing of neutral words were affected by the emotional type of VR contexts.

All in all, the analyses in Tables 1–3 combined answered Research Question 1: (1) positively valenced nouns were processed more relative to positively valenced adjectives under both positive and negative VR contexts. However, under positive VR context, negatively valenced adjectives were processed more relative to negatively valenced nouns, and neutral nouns were processed more as compared to neutral adjectives, which was different from negative VR context where insignificant differences were found between negatively valenced nouns and adjectives, and neutral nouns were found less processed that neutral adjectives; (2) both positive and negative VR contexts could stimulate participants to select more positive words though negatively valenced words were processed more in negative VR scenario that in positive VR scenario; (3) positive VR context could stimulate neutral words to be processed more similar to positive words, whereas negative VR could stimulate neutral words to be processed more similar to negative words.

### Analysis of galvanic skin responses under different virtual reality contexts

A 2 (context type: positive VR scenario and negative VR scenario) × 4 (galvanic skin response: relax baseline, after relaxation, negative baseline, and after negative experience) repeated-measures ANOVA was performed on galvanic skin responses under different VR scenarios (see Table 4). The main effect for galvanic skin response was significant (*F*_(3,339)_ = 39.95, *p* < 0.001, *η^2^* = 0.26), indicating significant different effects of different VR contexts on galvanic skin responses. Post-hoc multiple comparison showed significant differences between positive baseline and galvanic skin responses after positive VR experience (*T*_(114)_ = −5.94, *p* < 0.001, *M*_relaxed baseline_ = 1.74, SD = 1.42, *M*_after relax_ = 2.12, SD = 1.67), and between the negative baseline and the galvanic skin response after negative VR experience (*T*_(114)_ = 0.36, *p =* 0.72, *M*_negative baseline_ = 2.50, SD = 1.74, *M*_after negative experience_ = 1.95, SD = 0.18). Furthermore, there was a significant two-way interaction of VR context × galvanic skin response (*F*_(3,339)_ = 59.33, *p* < 0.001, *η^2^* = 0.34).

**Table 4:** Galvanic skin response under different VR contexts.

galvanic skin response	VR contexts	*M*	SD
Relaxed baseline	Positive context	1.72	1.53
	Negative context	1.75	1.33
After relaxation	Positive context	1.95	1.79
	Negative context	2.25	1.58
Negative baseline	Positive context	2.89	1.80
	Negative context	2.20	1.65
After negative experience	Positive context	3.54	2.01
	Negative context	1.64	1.95

The simple effect analysis also indicated differences in galvanic skin response under different VR contexts. (1) For relaxed baseline, no significant differences were found between the positive and negative VR scenarios in galvanic skin responses (*M*_positive_ = 1.72, SD = 1.53; *M*_negative_ = 1.75, SD = 1.33; *F*_(1,113)_ = 0.01, *p* = 0.91, *η^2^* = 0.001); (2) For galvanic skin responses after relaxation, no significant differences were found between the positive and negative VR scenario (*M*_positive_ = 1.95, SD = 1.79; *M*_negative_ = 2.25, SD = 1.58; *F*_(1,113)_ = 0.91, *p =* 0.34, *η^2^* = 0.008); (3) For negative baseline, significant differences were found between the positive and negative VR scenarios (*M*_positive_ = 2.89, SD = 1.80; *M*_negative_ = 2.20, SD = 1.65; *F*_(1,113)_ = 4.54, *p =* 0.035, *η^2^* = 0.039); For galvanic skin responses after negative experience, significant differences were found between the positive and negative VR scenarios (*M*_positive_ = 3.54, SD = 2.01; *M*_negative_ = 1.64, SD = 1.95; *F*_(1,113)_ = 35.07, *p <* 0.001, *η^2^* = 0.237).

Altogether, Table 4 answered Research Question 2, indicating that arousal intensity of negative emotions stimulated by negative scenarios was significantly greater than that of positive emotions by positive scenarios. Namely, compared to positive feelings induced by positive VR contexts, negative feelings induced by negative VR contexts were stronger. The different emotional intensity aroused by different valenced VR context provides online evidence to an individual’s different emotional responses under different emotional contexts. Additionally, these findings to some extent also provide physiological explanations for participants’ different offline behaviors in light of emotional words selections after their experiencing of different VR scenarios, because galvanic skin responses could indicate and influence an individual’s stimulated physiological and psychological responses (Kemeny, 2003; Bai and Yue, 2013).

## Discussion

### Effects of different virtual reality contexts on the processing of different valence

The increased processing of positively valenced words in positive VR context and negatively valenced words in negative VR context further adds to the growing literature that individuals were more likely to identify and process emotional words related to their own emotional experiences, confirming the situation-consistency effects in line with Meshi et al., 2016 and Kim (2012). These findings also confirmed the self-related mood-consistency effects that postulated lexical processing in the domain of valence was related to an individual’s mood, supplementing previous studies from VR simulated contexts (e.g., Pavlenko, 2008; Yue et al., 2020). Emotional cognition is closely related to embodied experience, and the accuracy and activation of emotional words processing are closely related to an individual’s emotional experiences (Fan et al., 2018). Therefore, an individual is more likely to process emotional words that are consistent with the situation (Zhang et al., 2012). Thus, the reinforcement of context interaction by VR technology explains above all the current findings that positive scenarios activated more positively valenced nouns processing, while negative emotional scenarios activated more negatively valenced words processing.

It is noteworthy that different from Tacikowski and Ehrsson (2016) who argued that the advantage of self-related information processing at the semantic and conceptual level was not obvious, the current study found that an individual’s embodied experience of emotional scenarios could affect their perception and processing of self-related emotional words. The different findings might be due to the different emotional contexts in Tacikowski and Ehrsson (2016) who used discrete emotional face task and in the present study that used VR created emotional context. Linguistic information forms through multiple sensory-motor interactions with the environment (Kolbeneval and Alexandrov, 2016). Virtual reality can provide participants a closer approximation and connectivity strength to a real-world emotional experiences, which can deepen an individual’s environment experience (Bian et al., 2015; Roy et al., 2019). Strong interaction can promote an individual’s feelings of emotional contexts to enhance strong emotional synergy (Wang, 2018; Zhang, 2019a; Zhang and Zhang, 2021). In addition, increased density of positive and negative information in memory can cause faster activation due to the connectionist models of memory (Barriga-Paulino et al., 2022). Therefore, the obvious emotional synergy found in the present study could be due to the reinforcement of context interaction by VR technology. The findings of this study, to a certain extent, also provide a reference for the exact match between forms, meanings, and conceptual features of an individual’s lexical processing.

It is also noteworthy to point out two interesting findings in the present study. One is that different from Li et al. (2020) who found that neutral information was stimulated more in positive than in negative contexts, the present study found that positive VR context could stimulate neutral words to be processed more similar to positive words, whereas negative VR could stimulate neutral words to be processed more similar to negative words consistent with Wieser et al. (2014). This finding further confirms that neutral information, due to its instability and ambiguity in valence, could be more influenced by valence of the contexts to show top-to-down processing tendency (Leleu et al., 2015). Another interesting finding is that although more negatively valenced words were processed in negative VR scenario relative to positive VR scenario, negative VR scenario was also found to be able to stimulate more positive words processing. Given the special characteristics of the participants in the present study, one possible explanation might be the forgettable nature of negative information, which has been always found for aged adults consistent with socioemotional selectivity theory (Charles and Mather, 2003). The present participants were all from a vocational school, suffering less from academic pressures than university students. Accordingly, the relevance of negative stimuli for attainment of goals could be less, and thus less attention would be paid to negative stimuli (Kousta et al., 2009). Therefore, the vocational students, similar to aged adults, tend to put greater investment in emotionally meaningful goals to avoid consciously or unconsciously negative information to select more positive words even under a negative context. However, due to the complex relationships between age and emotional words processing, more studies with different levels of participants are needed to verify this explanation.

### Effects of different virtual reality contexts on the processing of different word classes

The current study also found that under VR contexts, be it positive or negative, there were processing advantages of positively valenced nouns over positively valenced adjectives, whereas the processing differences for negatively and neutrally valenced nouns and adjectives varied according to different VR contexts: Positive VR could stimulate more processing of neutral nouns and negative valenced adjectives, whereas negative VR could stimulate more processing of neutral adjectives. In general, these findings confirmed that nouns and adjectives had different emotional activation and valence intensity due to their different specificity and imageability consistent with (Xia et al., 2016; Peti-Stantić et al., 2021). These findings also indicated rather complicated effects of different VR contexts on word classes, which could be mediated by words’ valences due to their different emotional arousal and processing mechanisms (Kolbeneval and Alexandrov, 2016; Zhang, 2019a; Yue et al., 2020).

Specifically speaking, the significant processing advantages of positively valenced nouns over adjectives in positive VR context is, to some extent, in congruence with Zhu et al. (2011) who found more processing of nouns as compared to verbs in positive context, irrespective of valences, confirming that nouns relative to adjectives could be processed more due to its high concreteness and high imageability. However, different from Zhu et al. (2011) who found that negative context could lessen the processing advantages of nouns, the present study also found processing advantages of positively valenced nouns over adjectives in negative VR context. The different findings might be attributed to the different counterparts in term of word class in the two studies because adjectives and verbs have different concreteness and imageability, indicating different processing difficulties in favor of verbs (Peti-Stantić et al., 2021).

Additionally, in line with Zhang (2019a,b) who found that negative situations could activate more processing of negative adjectives compared to negative nouns, the current study further suggested that positive situations could also activate more processing of negative adjectives compared to negative nouns. One possible explanation might be the enhanced simulation degree and concreteness of adjectives in VR context as compared to nouns whose concreteness has already reached its end (Zhang and Zhang, 2015). Emotion and experience interact with each other (Glenberg et al., 2009), and different depth of experience and embodiment can stimulate different processing (Sell and Kaschak, 2011). Different from Zhang (2019a,b) who used emotional words in a text as stimuli, the current study used VR simulated emotional context as stimuli, indicating, to some extent, enhanced emotional experience depth and people-environment interaction depth (Boroditsky and Ramscar, 2002), which, in turn, could elicit more concreteness and simulation degrees to adjectives, and consequently lessen the processing advantages of nouns while increasing the processing of adjectives.

The contradictory findings revealed in positive and negative VRs as to neutral nouns and adjectives adds the complexity of interactions between VR contexts, word classes and valences. Nouns and adjectives owning to their different neural mechanisms have different stability, consistency and hierarchy (Waxman and Booth, 2001; Kolbeneval and Alexandrov, 2016). Positive context could extend more of an individual’s attention and active thinking (Baumann and Kuhl, 2005), and according could promote more processing of nouns due to their higher concreteness and imageability, and lower difficulty relative to adjectives (Zhu et al., 2011; Peti-Stantić et al., 2021). Therefore, neutral nouns could be processed more than neutral adjectives because nouns were “conceptually related to entities that are less diffuse and have a spatial basis” while adjectives were “conceptual separation of the quality from the object it describes” (Peti-Stantić et al., 2021, p. 1809–1810). However, under negative context, the advantage of noun processing could be reduced because of mindfulness meditation (Zhu et al., 2011; Morgan and Jonah, 2021). Since our conclusion is based on VR contexts which is different from the laboratory settings, future studies are needed to shed more light on the relations between valence, word classes, and contexts.

### Galvanic skin responses and lexical processing under different virtual reality contexts

This study also found more galvanic skin responses in the negative VR context than in the positive VR context, providing to some extent physiological foundation to particpants’ negative bias in information processing (Charash and Mckay, 2002; Crossfield and Damian, 2021). The individual’s more psychological and behavioral responses to negative stimuli in negative VR contexts as compared to positive VR contexts is in line with the majority of the valence studies (Eilola et al., 2007; Kennedy and Most, 2015; Crossfield and Damian, 2021). Furthermore, the galvanic skin responses under VR simulated immersive context further verified the findings of Jay et al. (2008) and Kennedy and Most (2015), adding new evidence to the advantage of negative stimuli over positive stimuli in quantity, intensity, and neural responses. Additionally, the current study, based on VR, further illustrated that galvanic skin responses are important indicators of an individual’s responses under different stimuli (Bai and Yue, 2013).

The different galvanic skin responses may explain partly the different lexical processing under different VR scenarios. As mentioned above, be it a noun or an adjective, the processing of neutral words was found to be more than that of negatively valenced ones under the positive VR context, whereas under the negative VR context, the processing of the neutrally and negatively valenced words were found to be similar to each other. The relatively more processing of negatively valenced words and greater galvanic skin responses under the negative VR context as compared to the positive VR context might indicate close correlations between the two. Therefore, it is very likely for galvanic skin responses, together with VR contexts, to affect participants’ selection of words. Furthermore, the close correlations between words’ processing and galvanic skin responses also lend additional support to the situation-consistency effects, indicating that situational context could not only modulate an individual’s processing of emotional information (Diéguez-Risco et al., 2015; Lindquist, 2017) but possibly exert cumulative effects on an individual’s emotional information processing through galvanic skin responses. However, more research in this strand is called for, given the paucity of related evidences.

What needs our special attention is that inconsistent with Li et al. (2020) who found that the arousal of neutral information in the self-related context was significantly higher, this study found that negative contexts could induce and enhance more negatively valenced words processing as compared to neutral words and more galvanic skin responses. The differences may be attributable to the different stimulus types. Different from the emotional facial stimuli used by Li et al. (2020), the current study used VR simulated negative immersion context where stimulus intensity could be enhanced by the embodied cognitive experiences, and accordingly an individual’s perception and interaction of realistic situations (Roy et al., 2019). Therefore, the different findings may suggest that while an individual’s responses to stimuli are primarily related to emotional valence as compared to emotional intensity, it is perhaps confined to laboratory studies. As for lexical processing and galvanic skin responses under an enhanced context such as VR emotional context, emotional intensity may exert more effects than valence, because VR can create an enhanced self-related context to enhance an individual’s galvanic skin responses and emotion information processing (Bai and Yue, 2013; Meshi et al., 2016).

## Conclusion

The current study constitutes a new attempt to assess the influence of valence and word classes on lexical processing under different VR emotional contexts. In addition, it also investigates an individual’s galvanic skin responses under different VR contexts. Overall, the findings align with the proposal that incorporating immersive realistic and complex experimental environments through VR may provide a more enhanced interaction with contexts and, consequently a stimulated lexical processing of similarly valenced words and galvanic skin responses, confirming the repeated reports of the enhanced alignment in enhanced interaction with the context (Wang, 2018; Zhang and Zhang, 2021) from a dimension of emotion. Furthermore, the research into the relation between valence, word classes, and lexical processing in the context of VR emotional experiences extends the situation-consistency effects and the mood-consistency effects research, and provides new evidence that different valenced words’ processing could be mediated by different word classes, indicating that positive context, compared to negative context, could reduce the effects of word classes on valence in lexical processing.

In addition to the aforementioned theoretical significance, the current study also has important practical implications. First, the results showed different processing preferences for emotional words under different VR contexts, providing opportunities for further studies to intervene an individual’s experience of emotional context, and accordingly to better their emotional experiences and regulation (Ni and Jin, 2020). Second, the mediating effects of word classes on valence in lexical processing found in the current study supports earlier calls for more investigation of the interactions between word types, valence and context (El-Dakhs and Altarriba, 2019). Third, the significant processing differences between different word classes under different VR contexts also pave the way for further context research to address the processing of emotional words with different conceptual features.

The study results indicate the promise of linking physiological responses across different VR contexts and words with different valence and word classes. However, a few limitations resulting from design decisions indicate several interesting directions for future research. First, while the differences between nouns and adjectives had the benefit of examining the mediating effects of word classes on valence, these discrepancies might also restrict the direct mapping of concreteness and imageability between the two word classes (Altarriba and Bauer, 2004; Peti-Stantić et al., 2021). Second, word arousal and word types play an important role in emotional research and, to some extent, the most robust in lexical processing (Kazanas and Altarriba, 2016; El-Dakhs and Altarriba, 2019; Zhang et al., 2019), but this study did not differentiate the arousal of different word classes, neither did it distinguish the emotion-label from the emotion-laden. Therefore, follow-up research also needs to add these dimensions to further analyze the effects of word classes under different VR contexts. Additionally, it is also important to carry out further studies with gender-balanced participants to overcome the limitation of recruiting more female participants in the current study due to the gender structure of students in the School. Third, although the present study tried to control the role of semantic relatedness between the words to be selected and the VR scenarios to be experienced, it is inevitably that some of the words were more or less semantically related to the VR scenrios, which could influence the results of the task. Therefore, future research is called for to further gauge the effects of semantic relatedness on the processing of words under different VR scenarios. Last but not the least, due to the limitations of VR materials provided by our cooperative VR company, we could not find a more joyful VR scenario. Further studies should be done with a more joyful VR scenario to investigate whether there could be any differences for emotional words processing. In a word, much more research is needed to draw a comprehensive account of the relationships between emotion and experience.

## Author’s note

SZ, PhD, is Professor of Applied Linguistics in School of Foreign Studies, Zhejing Gongshang University, Hangzhou, China. Her major interests and 50-plus publications are on second language acquisition, second language processing, and corpus-based linguistics, with a strong focus on affective and psychological factors and teaching methodology in language teaching and learning. Email: suminzhang@zjgsu.edu.cn; hbsdzsm@163.com.

XW, is a senior lecturer of Wu’an Comprehensive Vocational Education Center, Wu’an, Hebei, China. Her major interests are on education, second language acquisition, and language teaching and learning.

XZ, is teaching assistant of Mental Health Education Center of Guizhou Forerunner College. His major interests are on mental health, behavioral science, and education.

## Data availability statement

The original contributions presented in the study are included in the article/supplementary material, further inquiries can be directed to the corresponding author.

## Author contributions

SZ conceived and designed the study, collected the data, drafted, revised the manuscript and got it ready for submission. XW and XZ helped in data collection, analyzed and revised the manuscript and got it ready for submission under the instruction of SZ. All authors contributed to the article and approved the submitted version.

## Funding

The project was supported with a research grant from the 14th Five-Year Plan Teaching Reform Project of Higher Education in Zhejiang Province (jg20220256), the School-level Graduate Education Reform Project (YJG2022104) and the Introduced Talents Project (1070XJ2322012), Zhejiang Gongshang University, Zhejiang, China, to SZ. SZ was also supported with a grant from Project of Discipline Innovation and Advancement (PODIA) from Foreign Language Education Studies, Beijing Foreign Studies University (grant number: 2020SYLZDXM011), Beijing, China.

## Conflict of interest

The authors declare that the research was conducted in the absence of any commercial or financial relationships that could be construed as a potential conflict of interest.

## Publisher’s note

All claims expressed in this article are solely those of the authors and do not necessarily represent those of their affiliated organizations, or those of the publisher, the editors and the reviewers. Any product that may be evaluated in this article, or claim that may be made by its manufacturer, is not guaranteed or endorsed by the publisher.

## Appendix 1

Positive nouns: 爱情(love) 才华(talent) 成就(achievement) 财富(fortune) 正义(justice) 诚意(sincerity) 冠军(champion) 笑容(smile) 光明(light) 花朵(flower).

Negative nouns: 厕所(toilet) 苍蝇(fly) 细菌(bacteria) 小偷(thief) 耻辱(shame) 恶意(spite) 苦难(suffering) 疯子(lunatic) 棺材(coffin) 魔鬼(devil).

Neutral nouns: 宫廷(palace) 国防(national defense) 河流(river) 家长(parents) 金融(finance) 列车(train) 心脏(heart) 边境(border) 差别(difference) 成年(adult).

Positive adjectives: 宏伟(magnificent) 有趣(interesting) 辉煌 (glorious) 坚强(strong) 活泼(lively) 热心(ardent) 珍贵(precious) 勇敢(brave) 旺盛(vigorous) 壮丽(magnificent).

Negative adjectives 卑贱(ignoble) 奸诈(falsehearted) 歹毒(vicious) 自私(selfish) 虚伪(hypocritical) 惨忍(merciless) 可耻(disgraceful) 凄凉(desolate) 悲哀(woeful) 残酷(cruel).

Neutral adjectives 单独(alone) 忙碌(busy) 沉默(silent) 急切(urgent) 零星(sporadic) 剧烈(drastic) 深沉(deep) 匆忙(hurried) 依旧(still) 夸张(exaggerated).
